# An Online Minimally Guided Intervention to Support Family and Other Unpaid Carers of People With Dementia: Protocol for a Randomized Controlled Trial

**DOI:** 10.2196/14106

**Published:** 2019-10-10

**Authors:** Ángel C Pinto-Bruno, Anne Margriet Pot, Annet Kleiboer, Rose-Marie Droes, Annemieke van Straten

**Affiliations:** 1 Department of Clinical, Neuro- and Developmental Psychology Amsterdam Public Health Research Institute Vrije Universiteit Amsterdam Amsterdam Netherlands; 2 School of Psychology University of Queensland Brisbane Australia; 3 Optentia Research Focus Area North-West University, Vanderbijlpark Gauteng South Africa; 4 Department of Psychiatry Amsterdam University Medical Centers (at VUmc) Amsterdam Public Health Institute Amsterdam Netherlands

**Keywords:** informal carers, dementia, ICT intervention, online, perceived stress

## Abstract

**Background:**

About three-quarters of people with dementia live in their own homes, with help from family members and/or other unpaid carers, such as friends or neighbors. Often, unpaid carers themselves experience negative consequences, such as stress, burden, and symptoms of depression or anxiety. Research has shown that these consequences can be alleviated by psychosocial and psychological interventions. Moreover, there are indications that those interventions can be effective when offered online.

**Objective:**

This paper describes the protocol of a randomized controlled trial (RCT) that will take place in the Netherlands to evaluate the effectiveness of iSupport, a minimally guided, internet-based intervention to improve carers’ mental health and coping resources.

**Methods:**

A superiority two-arm RCT comparing the effects of the online support program with a waiting list control condition will be carried out in the Netherlands. The iSupport intervention was developed by the World Health Organization and is based on cognitive behavioral therapy principles. It has five main themes divided into 23 lessons. Carers can pick and choose which lessons they want to complete. We aim to recruit 200 unpaid carers. The experimental group (n=100) will be provided with access to the intervention for 3 months following randomization; those in the waiting list control group (n=100) will be granted access to the intervention after 3 months. Assessments will be conducted at baseline (T0), 3 months after baseline (post intervention, T1), and 6 months after baseline (follow-up, T2). The primary outcome is perceived stress, measured by the Perceived Stress Scale. Secondary outcomes are symptoms of depression and anxiety, caregiver burden, sense of competence, self-efficacy, mastery, and carers’ attitudes toward dementia and their person-centered approach (ie, to what extent carers tailor the provided care to the interest, needs, and history of the person with dementia).

**Results:**

Recruitment for the trial started in January 2019. As of July 2019, we have enrolled 120 participants. Data collection is expected to be completed by March 2020. Once all the data have been collected, we will conduct the data analyses between April and May 2020. We aim to publish our results in a manuscript by June 2020.

**Conclusions:**

Online interventions have shown promising results in improving the mental health of carers of people with dementia. Additionally, online interventions may overcome accessibility barriers. If successful, this intervention will have important potential for implementation as a public health intervention, since costs and support by trained staff are minimal.

**Trial Registration:**

Netherlands Trial Register (NTL) NL6417; https://www.trialregister.nl/trial/6417

**International Registered Report Identifier (IRRID):**

DERR1-10.2196/14106

## Introduction

It is expected that the number of people with dementia will grow exponentially in the coming years; most of them will be cared for by family and other unpaid carers [1,2]. In Europe, families and other unpaid carers provide about 75% of the care or supervision for people with dementia living at home, including help with activities of daily living, finances, and arranging care such as scheduling appointments with professionals [3,4]. The provision of unpaid care often affects carers’ lives negatively. They are prone to feelings such as stress, depression, anxiety, and being overburdened when providing care for a prolonged period of time [5]. This is especially notorious in dementia where the person becomes increasingly dependent on care during the course of the disease [6].

Consequently, the World Health Organization (WHO) identified family members’ and other unpaid carers’ need of support as a key priority [4,7]. The Dutch government also framed the improvement of the quality of life of carers of people with dementia as a health priority. One of the objectives of the Dutch dementia strategy is to develop and implement new care models with effective tools and interventions for both people with dementia as well as their unpaid carers [8].

In recent years, several types of interventions have been developed to ameliorate the health and well-being of carers of people with dementia. Examples are psychoeducational programs, cognitive behavioral therapy (CBT), respite care, and occupational therapy [9]. Psychological treatments aimed at improving carers’ ability to cope with problematic behavior, to cope with stressful situations, to enhance communication with the person with dementia, and to ask for support from others have been demonstrated to be effective in improving carers’ mental health and well-being [10-12]. Despite the progress of psychological face-to-face interventions specifically designed for carers of people with dementia, most carers still do not use or are unable to access this professional support because they are unaware of its existence, they have time constraints, or they do not want psychological help (ie, negative attitude possibly reinforced by cultural influences) [7]. Online interventions may be an effective solution to overcome these accessibility barriers. Previous research has found promising results of online interventions even when compared to face-to-face interventions [13-18]. Unguided interventions are generally seen as less effective than those that are guided by a therapist or coach, whether online or by telephone [16]. However, unguided or minimally guided interventions have the advantage that they can be easily scaled up because no extensive input of a therapist is needed. Additionally, online interventions in any format are advantageous for people because they do not have to schedule specific appointments and they do not need to travel to any particular setting. Furthermore, online interventions might help reduce the stigma that still remains in society regarding dementia and receiving psychological support as a carer. Therefore, with the help of an international expert panel, the WHO has developed iSupport, an online knowledge and skills training program, which aims to improve carers’ mental health and coping resources. iSupport is based on psychoeducation and CBT techniques, such as activity scheduling, relaxation, and cognitive reframing. A first adaptation of iSupport has been carried out for India. A pilot study was carried out with the adapted English version in India from 2017 to 2018 [19]. For our study, we translated iSupport into Dutch and adapted the text in line with the WHO’s adaptation and implementation guide for the Netherlands.

Currently, families and other unpaid carers in the Netherlands can find information, training, and support through a wide variety of online internet resources. These resources include the following: informative websites (eg, dementie.nl, owned by the Alzheimer’s Association in the Netherlands); an online social support intervention [20]; free and paid apps to organize family care, organize other unpaid care, and increase peer support to improve communication between carers [21]; a self-guided, e-learning course with different levels of knowledge tailored to the needs of unpaid and professional dementia carers [18]; and a guided internet intervention to reduce psychological distress of caregivers of people with dementia with online coaching by a mental health professional [22,23].

iSupport is an addition to this broad spectrum of internet interventions. As an online training and support program with minimal guidance, iSupport focuses on mental health outcomes of family and other unpaid carers. The iSupport program is flexible (ie, carers can select the topics they prefer) and personalized (ie, provides automatically personalized feedback). This paper describes the design of a randomized controlled trial (RCT) to examine the effectiveness and usability of iSupport for family and other unpaid carers of people with dementia; we examined the tool’s ability to reduce carers’ distress and improve mental health (eg, depression and anxiety symptoms and caregiver burden) and to increase coping resources (eg, mastery, self-competence, and self-efficacy) compared to a waiting list control group.

## Methods

### Study Design

**Figure 1:**
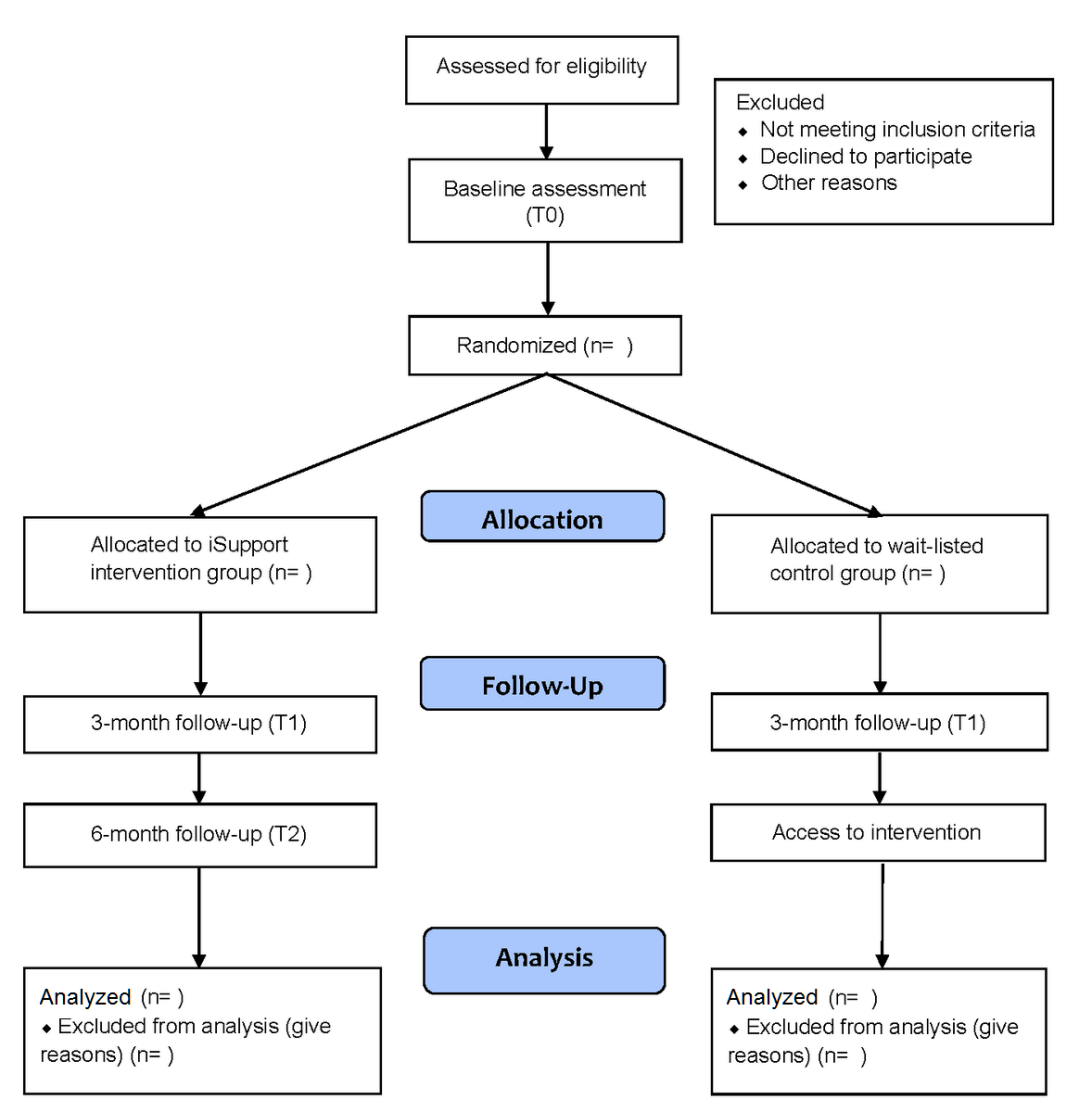
Consolidation Standards of Reporting Trials (CONSORT) diagram for this study.

The study has been designed as a two-arm superiority RCT. Carers will be randomized to (1) the intervention group that will use iSupport, an online training and support program, or (2) a waiting list control group. The iSupport intervention group will be provided with access to the intervention for 3 months immediately following the randomization; those assigned to the control group will receive access to iSupport 3 months after randomization (see Figure 1). The study will be carried out according to the Consolidation Standards of Reporting Trials (CONSORT) guidelines [24].

### Inclusion and Exclusion Criteria

The potential participants will be relatives or other unpaid carers of people with dementia. The participants must be at least 18 years old. We will focus on unpaid carers who are providing care to a family member, friend, or neighbor for at least 6 months, regardless of the number of hours per day they spend on care provision. To be included in the study, their well-being will need to have been affected at least to some extent. We will measure well-being via levels of stress, existence of depressive or anxiety symptoms, or experience of caregiver burden. This means that the carers will need to have a score greater than 13 on the Perceived Stress Scale (PSS) [25], a score of 4 or greater on the Hospital Anxiety and Depression Scale-Anxiety subscale (HADS-A) [26], or a score of 4 or greater on the Centre for Epidemiological Studies Depression scale (CES-D) [27]. Caregiver burden will be assessed using a one-item scale with a score ranging from 1 (no burden) to 10 (extreme burden); people will be eligible for the study if they scored 4 or greater.

Furthermore, there needs to be an indication that the person that is being cared for is indeed living with dementia. Therefore, carers will have to fill out the Ascertain Dementia 8-item informant questionnaire (AD8) [28]. The AD8 assesses the functional decline of the person with dementia attributed to cognitive impairment over the past years as reported by the carer. In order to be included, the score needs to be 2 or higher. This cutoff has been shown to have a good discriminative validity between people with and without dementia. We will also ask whether or not the person has been officially diagnosed with dementia, but this will not serve as an inclusion criterion.

We will exclude people who are unable to comprehend written Dutch or have no access to the internet. If both groups differ regarding the professional support they have received, we can control for it in the analyses, but receiving professional support will be not be used as an exclusion criterion.

### Recruitment of Participants and Study Procedure

People will be recruited through different sources. First, Alzheimer Nederland (AN), the Dutch Alzheimer’s national association, will place announcements about the study on their website, social media pages, and newsletter, among other places. Second, study banners will be placed on other relevant websites. Third, the research team will leave brochures and posters while visiting Alzheimer’s cafés and memory clinics. All of our study announcements will refer to our study website [29], which contains information about the study purpose and procedures. Those who are interested in participating will be able to register through this study website. We will then contact them via email where we will include a brochure with the information and a link to the online battery of baseline questionnaires, which includes the screening questions and the online written informed consent form. At this stage, we will fully inform potential participants about the aim and procedures of the study and will again ask for online informed consent. Based on the answers to the screening questions from the battery of baseline questionnaires, we will check the inclusion and exclusion criteria. Those who do not fulfil our inclusion criteria or do not provide informed consent will not be able to continue to fill out the remainder questionnaires of the baseline battery. They will receive an automated message explaining why they cannot participate in the study. We will stress that they can consult their general practitioner and AN at any time. Those who fill out the full battery of baseline questionnaires and fulfil the inclusion criteria will be included in the study and will be randomized. The outcome of the randomization will be communicated to participants via email. Participants randomized to the iSupport group will receive the intervention log-in details and they will have access to the intervention for 3 months. After the third month, participants will no longer have access to the intervention. Those who are randomized to the waiting list control group will be informed that they will receive log-in details for iSupport after they have completed the 3-month assessment. They will be free to seek any help they want in the meantime. The postintervention measurement and follow-up measurement will be sent via email 3 months and 6 months after randomization, respectively.

### Ethical Approval

The trial will be conducted according to Dutch and European legal requirements and standards as well as the Declaration of Helsinki (World Medical Association, 2013). The study protocol was reviewed by the Ethical Committee of the Faculty of Behaviour and Movement Sciences of the Vrije Universiteit Amsterdam (approval number: VCWE-2017-126); according to the review by the Medical Research Ethical Committee of the Vrije Universiteit medical center, this study does not fall within the scope of the Medical Research with Human Beings Act (review number: 2017.331).

### Randomization and Blinding

A randomization schedule with a 1:1 allocation ratio will be made with a computerized random number generator using variable block sizes of two and four by an independent researcher. We will stratify for gender and for the type of relationship between the carer and the person with dementia (ie, partner or other). After every inclusion, the same independent researcher will reveal the next randomization outcome so that allocation is concealed. Participants will be informed about the randomization outcome by email.

### Intervention: iSupport

iSupport is an online support program used to enhance self-care skills and support carers of people with dementia. The intervention has been developed by the WHO in collaboration with international experts in the field and Alzheimer Disease International. Final content decisions of the generic version were based on the outcomes of focus groups with professionals and unpaid carers, a pilot study (N=10) in India, and discussion within the project group. iSupport is based on the principles of CBT and includes techniques such as problem solving, relaxation, and cognitive reframing. During the development of the intervention, ethical principles and the needs of the carers were considered.

iSupport consists of 23 lessons distributed over five modules: (1) What is dementia? (one lesson), (2) Being a caregiver (four lessons), (3) Caring for me (three lessons), (4) Providing everyday care (five lessons), and (5) Dealing with challenging behavior (10 lessons). In Table 1, an overview of the topics covered through all modules and lessons is presented.

Carers may pick and choose which lessons they would like to do, depending on their needs and how relevant the topic of the lesson is to them. Every lesson follows the same format, consisting of information about the main topic of the lesson; small exercises, after which participants receive instant personalized automated feedback; and a summary of the lesson plus a relaxation exercise. In order to personalize the feedback and the content of the intervention, participants are asked to provide their own name, the name of the person with dementia, their relationship, and some other basic demographics (ie, gender and age). The program can be followed via the internet on a personal computer or tablet.

**Table 1:** Modules and lessons from iSupport.

Modules	Lessons
1. What is dementia?	1.1. Introduction to dementia
2. Being a caregiver	2.1. The journey together 2.2. Communication 2.3. Shared decision making 2.4. Involving others
3. Caring for me	3.1. Reducing stress in everyday life 3.2. Pleasant activities 3.3. Thinking differently
4. Providing everyday care	4.1. Eating and drinking: more pleasant mealtimes 4.2. Eating and drinking: preventing health problems 4.3. Toileting and continence care 4.4. Personal care 4.5. Enjoyable day
5. Dealing with challenging behavior	5.1. Introduction to challenging behaviors 5.2. Memory loss 5.3. Aggression 5.4. Depression and anxiety 5.5. Difficulty sleeping 5.6. Delusions and hallucinations 5.7. Repetitive behaviors 5.8. Walking and getting lost 5.9. Poor or decreased judgement 5.10. Putting it all together

Participants will be advised to use the iSupport program regularly to benefit as much as possible from their participation. It is anticipated that carers will be able to complete the whole program in 3 months. Participants will be able to indicate that they want some personal contact by sending their email address to our e-coaches. They will then be contacted by an e-coach three times via email: right after the first email, 1 month later, and 2 months later. Additionally, carers may contact the e-coach, if necessary, at any point of the intervention, although we aim not to have more than 10 interactions between e-coaches and participants. The purpose of the e-coach is to encourage participants to continue with the iSupport program, to explain anything that is not clear to the carer in the iSupport program, and to help to find additional support if needed. The emails that are sent by the users will be anonymized and used to improve the iSupport program. The coaches will be volunteers currently working for the telephone helpline of AN. They will complete a 4-hour training session by members of the research team in order to fully comprehend the intervention and the study.

The generic iSupport intervention is in English. However, during the developmental stages, attention has been paid to the possibility of adapting the intervention to different languages and cultural and economic situations. We adapted the generic version to the Dutch context. This means that the intervention has been translated by an official translator, who is knowledgeable in the field of dementia care, following the adaptation and implementation guide provided by the WHO. All text not pertinent to Dutch culture has been adapted (ie, names, activities, timetables, email addresses, etc). Afterward, the text was reviewed by staff of AN and an independent reviewer to ascertain that the adaptation was accurate and a dementia-friendly vocabulary was used.

### Intervention: Waiting List Control Group

Participants assigned to the waiting list condition will receive access to the iSupport intervention 3 months after the baseline session. We will stress that carers allocated to this group will be allowed to access any help they may need from online resources, to participate in support groups, or to access any type of professional help.

### Assessments

All assessments will be administered online and consist of a variety of self-reported measures. During the first measurement, we will assess the inclusion and exclusion criteria. Only those people that are eligible will complete the remainder of the baseline questionnaires. Table 2 provides an overview of the questionnaires and the number of items for each assessment.

### Primary Outcome

Our primary outcome is perceived stress. Perceived stress will be assessed with the PSS [25]. The PSS is a 14-item scale designed to measure global levels of stress and to assess to what extent participants perceive their lives as unpredictable, uncontrollable, and overloaded. Each item is scored on a Likert-type scale from 0 (never) to 4 (very often). The total score ranges from 0 (no stress) to 88 (very stressed).

**Table 2:** Overview of measures.

Collected data and instrument			Outcome measures	Items, n	T0^a^	T1^b^	T2^c^
**Demographic data of carers and persons with dementia**							
	Sociodemographic questions		Age, gender, etc^d^	15	x^e^	N/A^f^	N/A
**Inclusion criteria**							
	Burden scale		Caregiver burden	1	x	N/A	N/A
	AD8^g^		Functional decline	8	x	N/A	N/A
**Primary outcome**							
	PSS^h^		Perceived stress	14	x	x	x
**Secondary outcomes**							
	**Mental health**						
		CES-D^i^	Depression	20	x	x	x
		HADS-A^j^	Anxiety	7	x	x	x
		ZBI^k^	Caregiver burden	12	x	x	x
	**Mastery and sense of competence**						
		PMS^l^	Mastery	7	x	x	x
		RIS^m^ Elder Care Self-Efficacy Scale	Self-efficacy	10	x	x	x
		SSCQ^n^	Sense of competence	2	x	x	x
	**Attitude toward dementia and the care recipient**						
		ADQ^o^	Approaches to dementia	11	x	x	x
	**Treatment adherence, usage, and satisfaction**						
		SUS^p^	Usability	10	N/A	x	N/A
		Postlesson questions	Satisfaction	2 per lesson	N/A	x	N/A
		Analysis of platform data	Intervention usage	N/A	N/A	x	N/A

^a^T0: baseline assessment.

^b^T1: 3-month follow-up assessment.

^c^T2: 6-month follow-up assessment.

^d^Demographic data of carers: age, gender, marital status, number of children, children living at home, relationship with the person with dementia, level of education, months of caregiving, and average days per week providing care. Demographic data of persons with dementia: age, gender, marital status, living situation, and diagnosis.

^e^The questionnaire is included at the questionnaire battery for the different time points (T0, T1, T2), in opposition to N/A.

^f^Not applicable.

^g^AD8: Ascertain Dementia 8-item informant questionnaire.

^h^PSS: Perceived Stress Scale.

^i^CES-D: Centre for Epidemiological Studies Depression scale.

^j^HADS-A: Hospital Anxiety and Depression Scale-Anxiety subscale.

^k^ZBI: Zarit Burden Interview.

^l^PMS: Pearlin Mastery Scale.

^m^RIS: relational self-efficacy, instrumental self-efficacy, and self-soothing efficacy.

^n^SSCQ: Short Sense of Competence Questionnaire.

^o^ADQ: Approaches to Dementia Questionnaire.

^p^SUS: System Usability Scale.

### Secondary Outcomes

Our secondary outcomes are related to (1) mental health (ie, symptoms of depression, anxiety, and caregiver burden); (2) mastery and sense of competence; (3) attitude toward dementia and the care recipient (ie, person-centered approach); and (4) intervention usability, intervention use, and satisfaction with the content of the intervention.

Anxiety symptoms will be measured using the Dutch version of the HADS-A. The HADS-A [26] consists of seven items concerning anxiety complaints experienced in the past week. Subjects will score these feelings on a 4-point Likert scale from 0 (not at all) to 3 (nearly every day); the total score will range from 0 (no anxiety) to 21 (many anxiety symptoms).

Depressive symptoms will be measured with the CES-D [27]. This Likert-type scale consists of 20 items for which subjects will rate the frequency of symptoms during the past week. Scores range from 0 (rarely present or present none of the time: less than one day) to 3 (present most or all of the time: 5-7 days). The total score will range from 0 (no depression) to 60 (very depressed).

Perceived caregiver burden will be measured with the 12-item Zarit Burden Interview (ZBI) [30,31]. The scale measures a number of different types of burden, including the emotional, social, and financial impact of caring for someone. Each of the 12 items will be scored on a 5-point Likert scale from 0 (never) to 4 (nearly always). The total score will range from 0 (no burden) to 48 (very high burden).

Mastery will be measured using the 7-item Likert-type Pearlin Mastery Scale (PMS) [32,33]. The item scores range from 1 to 5, hence, the total scale score will range from 7 to 35. A high score represents internal mastery and indicates that someone has the feeling of being in control of situations. A low score represents external mastery and indicates that someone has the feeling that things are outside of their control.

Next, we will measure the feelings of self-efficacy with the relational self-efficacy, instrumental self-efficacy, and self-soothing efficacy (RIS) Elder Care Self-Efficacy Scale [34]. This scale measures the extent to which someone believes they are able to successfully master a specific task [35]. The scale consists of three subscales: (1) relational self-efficacy (three items): beliefs about one’s ability to maintain a cooperative and harmonious relationship with the recipient of care, (3) instrumental self-efficacy (four items): beliefs about one’s ability to accomplish tasks associated with the provision of personal care, and (3) self-soothing efficacy (three items): beliefs about one’s ability to maintain their own well-being in the midst of emotionally taxing and usually unrelenting demands. Each of the 10 items is rated on a 5-point Likert scale ranging from 1 (I’m certain I can’t do this) to 5 (I’m certain that I can do this). The subscale scores are derived by summing the item scores. Higher scores indicate increased self-efficacy.

We will measure sense of competence using the Short Sense of Competence Questionnaire (SSCQ) [36], which consists of seven items, five of which are already included in the ZBI. This means that we will ask participants to address the two remaining items. Three domains are distinguished in the SSCQ: (1) satisfaction with the person with dementia as a recipient of care, (2) satisfaction with one's own performance as a carer, and (3) consequences of involvement in care for the personal life of the carer. Each item is scored on a 5-point Likert scale, ranging from 1 (agree very strongly) to 5 (disagree very strongly). The total score will be derived by summing up the item scores.

The relationship between the carer and the person with dementia will be assessed with one of the subscales from the Approaches to Dementia Questionnaire (ADQ) [37]. This 11-item subscale measures *recognition of personhood*. This reflects the extent to which people have a person-centered understanding of dementia. It assesses to what extent the carer recognizes the care recipient as a unique and valuable individual. Each item will be answered on a 5-point Likert scale ranging from 1 (completely agree) to 5 (completely disagree). The total score will be a summation of the item scores and will range from 11 to 55. Higher scores will indicate more positive attitudes regarding personhood toward people with dementia.

We will use the 10-item System Usability Scale (SUS) [38] to evaluate the overall usability of the iSupport program. This questionnaire was specifically developed to evaluate information and communications technology products, websites, and applications. Each item is a statement and the respondent will need to indicate to what extent they agree with the statement on a 5-point Likert scale, ranging from 0 (strongly agree) to 4 (strongly disagree). An example of a statement is “I thought iSupport was easy to use.” The total score will range from 0 to 40 and will be multiplied by 2.5 to convert the scores to 0-100.

We will gather data about participant satisfaction from the completed lessons: after every lesson, carers will be asked two questions about how satisfactory the lesson was for them. The use of iSupport will be assessed by downloading track-and-trace data. We will examine the number of log-ins, the total time spent on the intervention, and the number of completed lessons.

Finally, we will also gather information on sociodemographic variables. For carers, we will collect information on the following: age, gender, relationship to the person they are caring for, civil status, level of education, average hours of care per week, and living arrangements (ie, cohabiting or not with the person they are caring for). For the person with dementia, we will collect information on the following: age, gender, existence of an official diagnosis, type of dementia, and time since the diagnosis was given.

### Sample Size

Perceived stress is our primary outcome measure and is used for the power calculation. Based on the results of a previous study about an online intervention for carers [39], we expect an effect size (Cohen *d*) of 0.40. Assuming an alpha of .05 and a statistical power (1-beta) of .80 in a two-tailed test, we will need 100 respondents in each of the conditions, resulting in a total of 200 participants. Calculation of the sample size was carried out with G*Power 3.1.9.2 (Heinrich-Heine-Universität Düsseldorf) [40].

### Statistical Analysis

We will first check whether or not the randomization was successful by comparing the two groups for all baseline variables with *t* tests for continuous data or chi-square tests for dichotomous data. We will also check for differences between study dropouts and those who remain in the study at 3 months. The primary analyses will be based on the intention-to-treat principle. Sensitivity analyses will be performed only with those participants that filled out the pre- and posttest questionnaires (ie, completers). Missing data handling will depend on the amount and pattern of missing data, selecting the most appropriate technique. We will use the generalized estimating equation to examine the association between changes in stress scores (ie, PSS scores) and treatment arms (ie, iSupport vs waiting list). We will correct for baseline scores if they are not equally distributed. We will examine the influence of age, gender, and relationship (ie, spouses vs other relationship) on the outcomes. These analyses will be repeated for burden, anxiety and depression, sense of competence, self-efficacy, and mastery. Next, we will express the between-group, posttest, effect sizes for the different outcomes using Cohen *d*. Cohen *d* is the difference between the two posttest means divided by the pooled standard deviation. An effect size of 0.8 is considered to be large, an effect size of 0.5 is considered to be moderate, and an effect size of 0.2 is considered to be small.

## Results

Recruitment for the trial started in January 2019. As of July 2019, we have enrolled 120 participants. Data collection is expected to be completed by March 2020. Once all the data have been collected, we will conduct the data analyses between April and May 2020. We aim to publish our results in a manuscript by June 2020.

## Discussion

In this paper, we outlined the design of an RCT to examine the effectiveness of iSupport for families and other unpaid carers of people with dementia in improving their mental health and coping strategies.

iSupport was developed by the WHO for global implementation and therefore includes guidelines for translation and cultural adaptation of the intervention. Internet interventions are a promising channel to prevent or treat mental health conditions of relatives and other unpaid carers who face greater risk of experiencing mental health problems themselves [41]. iSupport may increase unpaid carers’ worldwide accessibility to the support they need through the internet. An advantage of the intervention is that it is flexible and carers can decide which lessons they want to complete. Another advantage is that it can be provided with no or minimal professional support. This enables upscaling of the intervention without high costs. It might fit into a stepped-care model, in which only the carers who need more support are stepped up to face-to-face interventions. Additionally, family and other unpaid carers go through different stages during the course of dementia; their needs and preferences for training and support may substantially vary accordingly [42,43]. iSupport contributes to enriching and completing a continuum of interventions addressing the needs of carers in the Netherlands. This continuum should cover interventions from face-to-face to online interventions as well as guided to self-help interventions and everything in between.

The fact that the intervention is offered with minimal support might not only be a strength but also a weakness. It might lead to higher dropout rates. A review on the treatment adherence of online interventions carried out in 2008 [44] shows that in some trials participants never accessed the interventions tested, while others used them inconsistently. We try to minimize that risk by providing personalized automated feedback in iSupport as well as via periodic contact with e-coaches and the option to consult the e-coach more frequently. Hopefully, this encourages carers to continue with the intervention.

To the best of our knowledge, this is the first randomized trial for a minimally guided online intervention focusing on mental health outcomes for family and other unpaid carers of people with dementia in the Netherlands. This will provide valuable information for the Netherlands, but it will also contribute to the evidence for the use of iSupport at a global scale. An RCT of the Indian version of iSupport began in 2017 [19], and it is expected that more adaptations of iSupport will be completed worldwide in the future. We will test the effect of the intervention on different types of outcomes. Our study will try to overcome the possible challenges of RCTs of minimally guided online interventions, for example, by including personalized automated feedback. With our online and offline recruitment procedure, we expect to find a heterogeneous group of carers. Finally, the inclusion of a waiting list control group will allow all participants to benefit from the online tool.

In conclusion, using a two-arm RCT, our study will provide valuable information about the effectiveness of a minimally guided online intervention tool on participants by comparing outcomes with a waiting list control group. If the results of this study are satisfactory, iSupport may be a valuable response to an existing demand of carers for support in the Netherlands.

## Appendix

Multimedia Appendix 1 Peer-reviewer report from the European Commission.

## Abbreviations

AD8: Ascertain Dementia 8-item informant questionnaire
ADQ: Approaches to Dementia Questionnaire
AN: Alzheimer Nederland
CBT: cognitive behavioral therapy
CES-D: Centre for Epidemiological Studies Depression scale
CONSORT: Consolidation Standards of Reporting Trials
HADS-A: Hospital Anxiety and Depression Scale-Anxiety subscale
INDUCT: Interdisciplinary Network for Dementia Using Current Technology
ITN: Innovative Training Network
N/A: not applicable
PMS: Pearlin Mastery Scale
PSS: Perceived Stress Scale
RCT: randomized controlled trial
RIS: relational self-efficacy, instrumental self-efficacy, and self-soothing efficacy
SSCQ: Short Sense of Competence Questionnaire
SUS: System Usability Scale
T0: baseline assessment
T1: 3-month follow-up assessment
T2: 6-month follow-up assessment
WHO: World Health Organization
ZBI: Zarit Burden Interview
