# Supplementary material for: An Online Minimally Guided Intervention to Support Family and Other Unpaid Carers of People With Dementia: Protocol for a Randomized Controlled Trial
Source: JMIR Res Protoc. 2019 Oct 10;8(10):e14106. doi: 10.2196/14106 (PMC6819009; doi:10.2196/14106)
Supplement: Multimedia Appendix 1 [file resprot_v8i10e14106_app1.pdf]

Ref. Ares(2015)2011456 - 12/05/2015

# Proposal Evaluation Form

**EUROPEAN COMMISSION**

Horizon 2020 - Research and Innovation Framework Programme

**Evaluation Summary Report**

**Call:** H2020-MSCA-ITN-2015
**Funding scheme:** Training Networks
**Proposal number:** 676265
**Proposal acronym:** INDUCT
**Duration (months):** 48
**Proposal title:** Interdisciplinary Network for Dementia Utilising Current Technology (INDUCT)
**Activity:** MSCA-ITN-ETN: SOC

| N. | Proposer name | Country | Total Cost | % | Grant Requested | % |
|---|---|---|---|---|---|---|
| 1 | THE UNIVERSITY OF NOTTINGHAM | UK | 546,575 | 14.06% | 546,575 | 14.06% |
| 2 | UNIVERSITEIT MAASTRICHT | NL | 510,748 | 13.13% | 510,748 | 13.13% |
| 3 | KAROLINSKA INSTITUTET | SE | 527,318 | 13.56% | 527,318 | 13.56% |
| 4 | UNIVERSITY COLLEGE LONDON | UK | 546,575 | 14.06% | 546,575 | 14.06% |
| 5 | VRIJE UNIVERSITEIT BRUSSEL | BE | 501,120 | 12.89% | 501,120 | 12.89% |
| 6 | Charles University | CZ | 232,422 | 5.98% | 232,422 | 5.98% |
| 7 | WORLD HEALTH ORGANIZATION | CH | 265,226 | 6.82% | 265,226 | 6.82% |
| 8 | STICHTING VU-VUMC | NL | 510,748 | 13.13% | 510,748 | 13.13% |
| 9 | I MAS D y Empleo Serviconsulting S.L. (IDES S.L.) | ES | 247,872 | 6.37% | 247,872 | 6.37% |
| | Total: | | 3,888,604 | | 3,888,604 | |

**Abstract:**
Dementia raises complex challenges for people with dementia, their families, and society. The European Parliament has called for investment in high quality, innovative technology research to improve dementia care but so far there has been little benefit because research has generally been limited, small scale and methodologically flawed. In particular: the poor understanding between research and business of how people with dementia use technology means new applications are designed without an in-depth appreciation of people’s needs, preferences and limitations; there is little knowledge about practical, psychological and social barriers and facilitators to implementation making it hard to get results into practice; there is a serious shortage of research trained professionals who combine expertise on dementia care research and technology. In conjunction with INTERDEM the world’s largest network of psychosocial research for people with dementia this ITN will: develop a European multi-disciplinary, intersectoral educational research framework for Europe, to comprehensively train 15 early stage researchers (ESRs) to PhD level; provide the research evidence to show how technology may improve care and quality of life for people with dementia; and provide the European workforce with a much needed new generation of excellent research trained professionals. The objectives of INDUCT are to: determine practical, cognitive & social factors to improve usability of technology; evaluate the effectiveness of specific contemporary technology; trace facilitators & barriers for implementation of technology in dementia care. Using advanced methods in applied health research (e.g. randomised controlled trials, qualitative studies) INDUCT will provide the evidence needed to demonstrate how to make dementia care technology more usable, more effective, and better implemented in practice, culminating in an international consensus guideline for improving policy and practice.

## Evaluation Summary Report

### Evaluation Result

**Total score: 99.20% (Threshold: 70/100.00)**

### Form information

**SCORING**

*Scores must be in the range 0-5.*

***Interpretation of the score:***

- ***0–*** *The* ***proposal fails to address the criterion*** *or cannot be assessed due to missing or incomplete information.*
- ***1– Poor.*** *The criterion is inadequately addressed, or there are serious inherent weaknesses.*
- ***2– Fair.*** *The proposal broadly addresses the criterion, but there are significant weaknesses.*
- ***3– Good.*** *The proposal addresses the criterion well, but a number of shortcomings are present.*
- ***4– Very good.*** *The proposal addresses the criterion very well, but a small number of shortcomings are present.*
- ***5– Excellent.*** *The proposal successfully addresses all relevant aspects of the criterion.Any shortcomings are minor.*

### Criterion 1 - Excellence

Score: **5.00** (Threshold: 0.00/5.00 , Weight: 50.00%)

**Quality, innovative aspects and credibility of the research programme (including inter/multidisciplinary and intersectoral aspects)**
**Quality and innovative aspects of the training programme (including transferable skills, inter/multidisciplinary and intersectoral aspects)**
**Quality of the supervision (including mandatory joint supervision for EID and EJD projects)**
**Quality of the proposed interaction between the participating organisations**

*Strength(s)*

*+ The presented research project is highly innovative and significant. It brings together experts from different sectors in a meaningful way to improve dementia care.*
*+It is an excellent example of inter-sectoral research in terms of bringing together state of the art scientific knowledge and technology to result in innovations in practical applied settings.*
*+ The objectives of the proposal are clearly stated.*
*+The planned research projects are very clearly described and systematically build on each other.*
*+The proposed research has the potential to significantly increase the quality of life of a significant number of people in Europe, while the training will produce experts who can be future flagships of research in technological innovations in this research field.*
*+ The credibility of the proposed program is convincingly demonstrated.*
*+ The training programme is very well elaborated, clear and rich and apart from research skills and transferable skills is also a flexible system that addresses the individual ESRs special training needs. All these have the potential to train exceptionally well-educated professionals who have multiple high level skills.*
*+The network-wide training events are placed into the European Credit Transfer System.*
*+The interdisciplinary and intersectoral aspects of the programme are described in a detailed manner.*
*+ The role of the non-academic sector in the training programme is very well documented.*
*+ The training is very well designed and organised including three levels of skills training: dementia and dementia care research related skills (Level 1), transferable competences (level 2) and intersectoral expertise (level 3).*
*+ What is important, the training program covers such issues as end of life and long-term palliative care and topic concerning dignity of people with dementia.*
*+ The training will provide ESRs with entrepreneurial skills, knowledge on national dementia strategies, ethical issues and the technology use in dementia.*
*+ There is an excellent quality assurance process inbuilt into the supervision.*
*+Supervisors have a very good track record of experience with doctoral students.*
*+ Qualifications and expertise of the supervisors, as well as joint supervision arrangements, are in line with the aims of the project; they are presented in a detailed and well-structured manner.*
*+ The quality of supervision is very high, the host institution are top rank universities, one of them awarded by Alzheimer's society for the research in dementia.*
*+ The joint supervision is envisaged - ESRs will be assigned a primary supervisor at the host institution, third supervisor from another academic centre, the team of supervisors will include also a mentor from secondment (non-academic institution).*
*+ The inclusion of partner organizations is carefully composed and they offer highly relevant collaboration with the ESRs research projects e.g. the participation of the World Federation of Occupational Therapist Association and its offer to provide access to occupational therapists all over the world makes it possible to get substantial amount of data for several research projects.*
*+The synergies among participants are presented in a detailed and convincing way especially with several international networks.*
*+ The proposed interaction between participating organizations meets the current standards for a project focused on technological solutions.*
*+ The involvement of non-academic partners is high as each ESR will be exposed to non-academic environment (two secondment lasting 3 months each in two non-academic partners*

*-Weaknesses*
*no weaknesses identified*

**Overall comments**

*An excellent proposal taking adequately into consideration all aspects of quality of the research and training programme, the supervision activities and the interaction between participating organisations.*

## Criterion 2 - Impact

Score: **5.00** (Threshold: 0.00/5.00 , Weight: 30.00%)

**Enhancing research- and innovation-related human resources, skills, and working conditions to realise the potential of individuals and to provide new career perspectives**
**Contribution to structuring doctoral / early-stage research training at the European level and to strengthening European innovation capacity, including the potential for:**
**a) meaningful contribution of the non-academic sector to the doctoral/research training, as appropriate to the implementation mode and research field**
**b) developing sustainable joint doctoral degree structures (for EJD projects only)**
**Effectiveness of the proposed measures for communication and dissemination of results**

*Strength(s)*

*+ It is convincingly demonstrated that ESRs will acquire a special and much wanted cross-disciplinary and cross-sectional knowledge in public health, in the care sector and industry. This will greatly contribute to their employability both in academic and non-academic contexts.*
*+The proposed programme has a great potential to successfully enhance both research and applied skills including entrepreneurial skills of ESRs.*
*+ The proposed research will have significant impact on the ESRs career prospects due to interdisciplinary training enabling them to acquire knowledge concerning technology in dementia.*
*+ The training program will increase their employability outside academia i.e. in the industry sector.*
*+ The inter-sectorial collaboration will increase the understanding of commercial and technology aspects of dementia care.*
*+ The proposed programme may serve as best practice example how to structure an interdisciplinary and intersectoral research training in such specialized field like dementia care.*
*+ It is very convincingly demonstrated that the proposed technical developments may significantly increase European innovation capacity.*
*+ Due to the rich interconnection between the academia and the industrial sector there is a very high potential that technological devices are produced that contribute significantly to the European innovation arena.*
*+The non-academic sector is carefully selected and perfectly fits the research and training needs.*
*+ Due to its specific aims, the project is likely to enhance the contribution of the non-academic sector to doctoral/early research training.*
*+ The INDUCT project will be the first one to combine training of early stage researcher in the field of dementia and technology and enterprise.*
*+ The proposed measures for communication and dissemination of results meet the current standards.*

*+ Numerous and well-planned communication activities are foreseen.*
*+ Direct public engagement is envisaged.*
*+ A website involving a public part will be set up.*
*+ Scientific dissemination plans are adequate and reasonable including scientific articles and conference presentations.*
*+ Exploitation of results and intellectual property are sufficiently addressed.*

*- Weakness(es)*
*no weaknesses identified*

**Overall comments**

*The potential of the project to contribute to the development of innovation related human resources skills and to provide new career perspectives as well as its contribution to the structuring of doctoral / ESR Training at EU Level, and the effectiveness of proposed measures for communication and dissemination of results have been convincingly demonstrated.*

## Criterion 3: Implementation

Score: **4.80** (Threshold: 0.00/5.00 , Weight: 20.00%)

**Overall coherence and effectiveness of the work plan, including appropriateness of the allocation of tasks and resources (including awarding of the doctoral degrees for EID and EJD projects)**
**Appropriateness of the management structures and procedures, including quality management and risk management (with a mandatory joint governing structure for EID and EJD projects)**
**Appropriateness of the infrastructure of the participating organisations**
**Competences, experience and complementarity of the participating organisations and their commitment to the programme**

*+ Strength(s)*

*+ The work package objectives and IRPs are clear, coherent and well structured,*
*+ The allocation of tasks is functional and is in accordance with the research and training goals.*
*+ The presented deliverables are realistic, the description of the Lists of Deliverables documents the coherence and effectiveness of the workplan.*
*+ The timing of the different tasks within the workpackages are consistent with the research and training goals*
*+ A three-level clear management structure is presented with an appointed Ombudsperson and an Equality Advisor, the latter demonstrating the applicants' commitment to excellence and equal opportunities.*
*+There is a clear distribution of roles and responsibilities within the Management Committee.*
*+The recruitment policy is carefully composed to enable the supervisors' team to choose the best possible candidates (e.g. a training combined with interviews and candidates' presentations).*
*+ The recruitment plan takes multiple aspects of equal opportunity into consideration e.g. places special emphasis on reaching out and advertising the ESR positions in underrepresented regions of Europe.*
*+ Risk management and Intellectual Property Rights are addressed according to the current standards.*
*+ Gender aspects are sufficiently addressed.*
*+ Data management plan has been developed.*
*+ The infrastructures of all participating beneficiaries and partners are excellent, given the aims of the project and the needs of the fellows.*
*+The participating beneficiaries and partners build up a team of experts who can approach dementia from multiple highly relevant perspectives, their expertise is complementary and functional and intertwined.*
*+The participating organizations and partners are highly committed.*

*-Weakness(es)*
*- There is no clear schedule presented for the dissemination of the research results to the general public.*

**Overall comments**

*Overall coherence and effectiveness of the work plan, appropriateness of the allocation of tasks and resources have been very convincingly demonstrated. Management structures and procedures as well as infrastructures of participating organisations are highly adequate.*

## Operational Capacity

Status: **Operational Capacity: Yes**

*Not provided*

## EU funding to International / Third Country Organisations

**In the case of a participating international organisation, or in the case of a participating legal entity established in a third country not listed in the Annex I of the Work Programme, please confirm whether the participation is deemed essential for carrying out the action (mention the short name(s) of the participant(s) concerned).**

*Should this proposal be retained for funding, the participant World Health Organisation may exceptionally be granted the requested EU funding.*
